# Detection of Feigned Impairment of the Shoulder Due to External Incentives: A Comprehensive Review

**DOI:** 10.3390/diagnostics16020364

**Published:** 2026-01-22

**Authors:** Nahum Rosenberg

**Affiliations:** Sheltagen Medical, P.O. Box 164, Atlit 30300, Israel; nahumrosenebrg@hotmail.com; Tel.: +972-54-4685130; Fax: +972-4-8590930

**Keywords:** malingering, shoulder joint, secondary gain, symptom validity, motion analysis, biomechanics, electromyography, inertial sensors, artificial intelligence

## Abstract

**Background:** Feigned restriction of shoulder joint movement for secondary gain is clinically relevant and may misdirect care, distort disability determinations, and inflate system costs. Distinguishing feigning from structural pathology and from functional or psychosocial presentations is difficult because pain is subjective, performance varies, and no single sign or test is definitive. This comprehensive review hypothesizes that the systematic integration of clinical examination, objective biomechanical and neurophysiological testing, and emerging technologies can substantially improve detection accuracy and provide defensible medicolegal documentation. **Methods:** PubMed and reference lists were searched within a prespecified time frame (primarily 2015–2025, with foundational earlier works included when conceptually essential) using terms related to shoulder movement restriction, malingering/feigning, symptom validity, effort testing, functional assessment, and secondary gain. Evidence was synthesized narratively, emphasizing objective or semi-objective quantification of motion and effort (goniometry, dynamometry, electrodiagnostics, kinematic sensing, and imaging). **Results:** Detection is best approached as a stepwise, multidimensional evaluation. First-line clinical assessment focuses on reproducible incongruence: non-anatomic patterns, internal inconsistencies, distraction-related improvement, and mismatch between claimed disability and observed function. Repeated examinations and documentation strengthen inference. Instrumented strength testing improves quantification beyond manual testing but remains effort-dependent; repeat-trial variability and atypical agonist–antagonist co-activation can indicate submaximal performance without proving intent. Imaging primarily tests plausibility by confirming lesions or highlighting discordance between claimed limitation and minimal pathology, while recognizing that normal imaging does not exclude pain. Diagnostic anesthetic injections and electrodiagnostics can clarify pain-mediated restriction or exclude neuropathic weakness but require cautious interpretation. Motion capture and inertial sensors can document compensatory strategies and context-dependent normalization, yet validated standalone thresholds are limited. **Conclusions:** Feigned shoulder impairment cannot be confirmed by any single test. The desirable strategy combines structured assessment of inconsistencies with objective biomechanical and neurophysiologic measurements, interpreted within the whole clinical context and rigorously documented; however, prospective validation is still needed before routine implementation.

## 1. Introduction

Injury to the shoulder is a common cause of pain and disability, but not all reported shoulder limitations are genuine. Feigned restriction of shoulder joint movement refers to a patient’s deliberate exaggeration or fabrication of movement limitations for secondary gain, such as financial compensation, avoidance of work or military duty, or acquisition of medications [1]. The issue of symptom exaggeration and intentional feigning of musculoskeletal impairment has garnered increasing clinical and medico-legal attention. Recent analyses suggest that probable malingering and symptom magnification occur in approximately 29% of personal injury claims and 30% of disability-related evaluations, underscoring the significance of this phenomenon across diverse medicolegal contexts [2].

Malingering is not a medical diagnosis but rather a medicolegal concept indicating conscious deception; it must be distinguished from conditions like conversion disorder (functional neurological symptom disorder) or factitious disorder, in which symptoms are not produced for concrete external rewards [3]. Detecting malingering is critically essential: false claims of shoulder disability can misdirect medical resources, undermine trust, and inflate healthcare and insurance costs. At the same time, clinicians must approach the task cautiously, as false accusations can damage the patient–provider relationship and lead to legal or ethical issues [4].

**Figure 1:**
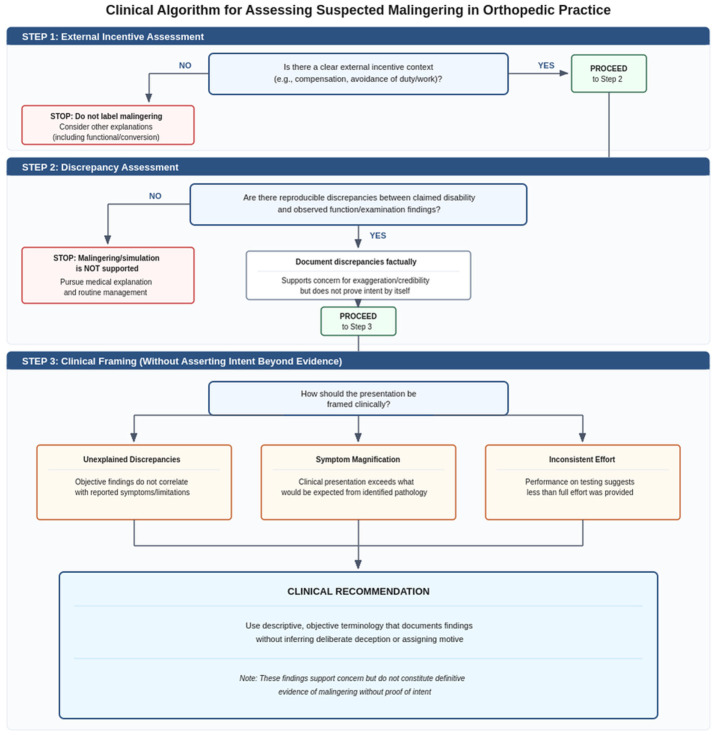
Conceptual flow chart for differentiating malingering (“simulation”), symptom magnification/exaggeration, abnormal pain response (APR), and functional/conversion-type presentations in shoulder disability assessment [1,2,3,4]. The schematic emphasizes that malingering is defined by intentional symptom production or exaggeration for external incentives and should be reserved for cases in which an external-incentive context and convergent evidence consistent with intentional deception coexist, whereas discrepancies and non-organic behaviors alone should be documented as validity indicators and may also reflect APR or non-malingering functional/conversion-type presentations [1,2,3,4].

To avoid conceptual and ethical ambiguity, we distinguish entities that may appear clinically similar but differ in intentionality and medicolegal meaning. Malingering (simulation) is a medicolegal concept defined as the intentional production or exaggeration of symptoms for external incentives; therefore, isolated “non-organic” signs should be documented as indicators of validity and interpreted cautiously rather than treated as proof of deception [1,2,3]. Symptom magnification and exaggeration describe overstated symptoms or disability behavior without necessarily establishing intent, and abnormal pain response (APR) in compensable shoulder injuries is best understood as an observed pattern of disproportionate or incongruent pain behavior that may reflect psychosocial factors and does not, by itself, establish malingering [4] (Table 1). To improve conceptual clarity and support ethically cautious reporting, the practical distinctions between these entities should be approached didactically, using a structured, logical decision-making approach (Figure 1). Given the ethical and legal consequences, documenting the observed discrepancies and their context rather than categorizing them is necessary, and reserving “malingering/simulation” for cases that meet its defining elements [1,3].

The true prevalence of feigned musculoskeletal symptoms is difficult to ascertain; however, studies in both civilian and military populations suggest that it is a significant minority of cases. In a cross-sectional analysis of 1000 workers’ compensation claimants with shoulder injuries, approximately 9% demonstrated an abnormal pain response (APR) indicative of non-organic behavior (e.g., exaggerated pain behaviors, inconsistent examination findings) [4]. These patients had higher self-reported disability and psychological distress than those without APRs. In the military, where malingering might be a punishable offense, self-reported rates of exaggeration are similar: approximately 6% of U.S. Army soldiers anonymously admitted to exaggerating or overreporting an injury for one year [5]. In civilian medicolegal assessments, even higher rates have been reported with specialized testing; for example, one report estimated that up to 29% of personal injury claimants showed evidence of feigning or exaggeration on rigorous psychometric evaluation [2]. These figures underscore that the phenomenon is common enough to warrant robust detection strategies.

Identifying feigned shoulder limitation is inherently challenging. Pain is subjective, and genuine shoulder conditions (such as rotator cuff tears or adhesive capsulitis) can produce variable exam findings and high levels of pain that might appear “non-organic” at first glance. Moreover, no single clinical sign or test can definitively confirm malingering [6]. Classic “non-organic” signs (such as Waddell’s signs, described initially in low back pain) were once thought to indicate malingering. Still, evidence shows they cannot reliably discriminate pure malingering from psychological factors or exaggerated-but-real pain [7]. It should be mentioned that Waddell’s signs, which are a set of observations like superficial tenderness or pain on axial loading, correlate more with poor outcomes and psychosocial issues than with outright faking. This means that a patient might have inconsistent shoulder exam findings due to psychological overlay or poor effort without intentionally lying, a distinction that complicates the evaluator’s job.

Recently, there have been significant advances in tools and methodologies for objectively assessing shoulder function, many of which can aid in malingering detection. Clinicians now have access to more quantitative approaches, including isometric dynamometry for strength testing [8], high-resolution imaging, motion capture systems, and even artificial intelligence algorithms that were not widely available or affordable previously. Research in forensic rehabilitation and sports medicine has yielded new protocols to test the sincerity of effort. For instance, specialized strength ratios and metrics have been developed that reveal distinct patterns, with an 80% sensitivity for detecting intentional submaximal effort in shoulder isokinetic force generation [9]. Wearable sensors and markerless motion tracking enable detailed analysis of shoulder kinematics during everyday tasks, providing an additional layer of evidence when a patient claims they “cannot lift the arm” but can do so under different conditions. Additionally, interdisciplinary collaboration (e.g., between orthopedic specialists, physiatrists, psychologists, biomechanists, and data scientists) has enriched the field of malingering detection, bringing techniques such as electromyography (EMG) and machine learning to bear on the problem from new angles.

Given the breadth of this topic, this review provides an integrated overview of detection methods for feigned shoulder movement restriction, focusing on recent advances, and relates these to strategies that might be tailored for military and civilian contexts, as the motivations and implications of malingering differ between these settings. The objective of this comprehensive review is to synthesize current evidence on methods for detecting feigned restriction of shoulder joint movement due to secondary gain, integrating clinical examination findings with objective biomechanical, neurophysiological, and emerging technological approaches, and to provide practical recommendations for clinicians and medicolegal evaluators. This review addresses the hypothesis that while no single test can definitively confirm feigned shoulder limitation, the systematic integration of clinical examination, objective biomechanical and neurophysiological testing, and emerging technologies can improve detection accuracy and provide defensible medicolegal documentation.

## 2. Methods

This comprehensive review synthesized current evidence on the detection of feigned shoulder joint movement restrictions due to secondary gain in clinical and medicolegal contexts. A systematic literature search was performed in the PubMed database using combinations of the following key terms: “shoulder movement restriction,” “malingering,” “symptom validity,” “functional assessment,” and “secondary gain.” A manual examination of the reference lists of relevant studies supplemented the search strategy.

Study selection prioritized investigations that reported objective or semi-objective measures of shoulder motion and muscle activation, such as goniometric assessments, surface electromyography, motion capture analysis, inertial sensors, and imaging-based quantification, with specific attention to experimental and clinical studies that distinguished genuine from feigned impairment. Preference was given to publications from the last decade (2015–2025) that employed validated diagnostic or biomechanical methodologies. Foundational works elucidating the conceptual or psychometric aspects of deception and effort testing were included, regardless of their date, due to their continuing relevance.

A narrative synthesis framework was employed to integrate heterogeneous findings across biomechanics, clinical orthopedics, and neuropsychology. The analysis emphasized interpretive integration of diagnostic validity indicators and behavioral markers over statistical meta-analysis, aligning technological advances, such as motion analysis algorithms, computer vision, and artificial intelligence–assisted pattern recognition, with established clinical examination practices. This approach facilitated the development of pragmatic clinical recommendations and outlined evidence-based directions for future multidisciplinary research in detecting simulated shoulder dysfunction.

A structured screening process was applied. PubMed records retrieved with the prespecified term blocks were screened by title and abstract, then full text, with additional studies identified through reference-list screening. This review is a narrative (non-systematic) synthesis intended to integrate heterogeneous evidence across clinical, biomechanical, neurophysiological, imaging, and medicolegal domains; therefore, despite structured searching and screening, some selection bias remains possible, and conclusions are interpreted cautiously.

To improve reproducibility and reduce biomechanical search bias, the PubMed strategy was expanded to include validity terminology in addition to shoulder terms. Alongside “shoulder or upper extremity,” “range of motion,” and “strength,” constructs such as “effort testing,” “sincerity of effort,” “submaximal/non-credible performance,” “performance/symptom validity,” “feigned pain,” “volitional exaggeration,” “symptom exaggeration,” “malingering/feigning,” and medicolegal contexts (e.g., “workers’ compensation,” “independent medical examination,” “forensic/medicolegal”) were added, with backward reference screening to capture relevant psychological literature. Studies were eligible if they addressed detection or interpretation of non-credible musculoskeletal limitation (shoulder-specific or transferable upper-limb paradigms); unrelated topics were excluded. Given heterogeneity and the lack of a gold standard, we did not exclude by design; instead, low-quality evidence (e.g., small/unblinded samples, instructed or feigning proxies, weak reference standards) was explicitly flagged and down weighted. Case reports/series were not used for diagnostic-accuracy claims and were included only, if at all, as illustrative examples.

## 3. Techniques of Clinical Detection

The detection of feigned musculoskeletal dysfunction requires a comprehensive, multidimensional approach. Since no single diagnostic test provides definitive evidence [10], current expert consensus emphasizes integrating detailed clinical examination findings with one or more objective assessment modalities to enhance diagnostic accuracy and validity.

### 3.1. Clinical Examination Tests

#### 3.1.1. Observation and Inconsistency

The clinical exam remains the first line of defense against malingering. Clinicians are trained to observe whether a patient’s reported symptoms and behaviors are congruent with known medical conditions. Key red flags include disproportionate pain behaviors (e.g., grimacing, groaning at minimal stimuli), inconsistencies between observed function and formal testing, and findings that defy anatomical or physiological patterns. In the shoulder exam, a patient feigning restriction might, for example, claim inability to actively raise the arm but can be seen using the arm normally when distracted (such as removing a shirt or reaching for an object when not thinking of being observed). Inconsistency between reported symptoms and objective findings is often cited as suggestive of malingering [11]. In the context of compensable shoulder injuries, the phenomenon of Abnormal Pain Response (APR) is characterized by a constellation of clinical features that may include inconsistent or non-reproducible range of motion (ROM), non-anatomical sensory disturbances, and disproportionate or exaggerated pain behaviors such as moaning, grimacing, or withdrawal during examination. These findings are considered indicators of symptom magnification or non-organic pain behavior rather than objective pathology. There is a report showing that approximately 9% of patients demonstrated Abnormal Pain Responses (APRs), and this subgroup exhibited greater levels of self-reported disability and a higher prevalence of psychosocial comorbidities. These findings suggest that non-organic pain behaviors are frequently associated with complex psychosocial factors, and in some cases may reflect elements of conscious symptom exaggeration [4].

The ‘red flags’ and ‘inconsistencies’ are used as validity indicators, not as proof of simulation. In practice, inconsistency is assessed by triangulating the reproducibility and physiological credibility of the discrepancy across tasks and time, context effects (e.g., marked improvement during distraction or spontaneous functional activities), and concordance with objective findings (e.g., imaging and, where indicated, EMG/NCS and quantified effort testing). Pain mediated by central mechanisms, kinesiophobia, and illness behavior can also produce variable performance and protective guarding; therefore, isolated inconsistencies should be described factually and framed as limitations in data interpretability. A medicolegal opinion suggesting feigned restriction should be reserved for cases in which multiple independent discrepancies converge and alternative explanations have been explicitly considered [12].

Several specific provocative maneuvers and distraction tests can be used to assess the sincerity of effort. A classic example is the “drop arm test”, which is normally used to detect rotator cuff tears but can also expose inconsistencies. When a patient reports profound weakness, the examiner can passively elevate the arm and instruct the patient to maintain the position. A patient feigning paralysis may allow the arm to drop abruptly; however, an involuntary deceleration or self-protective catching of the limb before impact suggests preserved motor control inconsistent with the claimed deficit [12,13]. Another approach is Hoover’s sign, described initially for leg weakness, which relies on the principle of complementary opposition: voluntary activation on one side elicits involuntary activation on the other side in neurologically intact pathways [13,14]. A prospective study of patients presenting with suspected stroke found that, in patients with leg weakness, a positive Hoover’s sign had a sensitivity of ~63% and a specificity of 100% for diagnosing functional (non-organic) weakness versus organic causes [14]. Analogous tests exploiting cross-body synkinesis have been developed for the upper limb, including the abduction finger sign and elbow flex-ex sign [15]. It should be noted that although several of the discussed diagnostic techniques reported high sensitivity or specificity, these values must be interpreted with caution due to the limited sample sizes in some studies. Furthermore, in several investigations, examinations were performed or interpreted by unblinded assessors, which introduces a potential source of observer bias. To ensure more reliable and generalizable findings, future validation studies should incorporate blinded assessment protocols to minimize interpretive bias and enhance methodological rigor [15].

Overall, distraction-based examination is supported by evidence that functional motor symptoms often improve with distraction and worsen with attention. Repeating shoulder movements while the patient is engaged in another task (e.g., turning the torso or head, or performing a functional maneuver such as undressing) can reveal a greater, more natural range of motion than during direct testing, indicating a non-organic component [16].

However, it is important to note that the presence of non-organic signs is not substantial proof of malingering. They simply indicate a mismatch between symptoms and objective pathology, which could be due to conscious faking, psychological factors amplifying pain perception, or a combination. Thus, clinical signs should be used to trigger further objective testing [7].

#### 3.1.2. Strength Testing and Coefficient of Variation

Manual muscle testing (MMT) is an unreliable method for assessing the sincerity of effort because patients can intentionally “give way” or produce submaximal contractions; prediction errors of MMT have been reported to reach 24% [17]. Objective strength testing using dynamometry improves quantification but remains effort-dependent. To evaluate effort consistency, functional capacity evaluations often examine the coefficient of variation (CV) across repeated maximal trials: consistent maximal efforts tend to show low variability, whereas inconsistent or intentionally reduced efforts may show higher variability. Although a CV above ~15% is sometimes used as an indicator for inconsistent effort, ROC analyses across CV thresholds (2.5–22%) indicate limited standalone diagnostic utility; at a 15% cut-off, sensitivity was found as modest (0.55) with only moderate specificity (~0.74), and no single CV threshold reliably discriminates maximal from submaximal performance [18]. Accordingly, CV should be interpreted as a supportive validity indicator and integrated with replication, contralateral comparisons, and other objective findings rather than used as a decisive criterion.

#### 3.1.3. Serial Examinations

An additional clinical strategy involves conducting repeated examinations over time [19]. A patient feigning a fixed limitation may not remember the exact degree of limitation they demonstrated previously, or fatigue of acting may lead them to exhibit a greater range later. Inconsistent performance between visits or between morning and afternoon sessions can be documented. Consistency is a hallmark of true pathology; for instance, a true adhesive capsulitis will exhibit the same limitation every time, both in active and passive motion. A malingered restriction might show full passive motion (if the patient relaxes when they think it is not being measured) but limited active motion, or inconsistent effort on passive testing. Clinicians might also employ the element of surprise. If greater movement is observed in a less formal context, it raises the question of the limitation.

Therefore, clinical tests rely on identifying internal inconsistencies and behaviors that are incompatible with known medical conditions. They set the stage for further evaluation. When clinical suspicion of feigned shoulder limitation is high, more objective testing modalities are then used to gather more conclusive evidence [20].

### 3.2. Imaging Techniques

Medical imaging is primarily used to verify the presence or absence of structural pathology that could explain a patient’s symptoms. In the context of detecting feigned shoulder disability, imaging plays a supporting role: finding clear evidence of a normal, healthy shoulder (or discrepancies between reported pain and imaging findings) can support the case that limitations are non-organic. However, the absence of evidence is not absolute proof of malingering. Many genuine pain conditions (like bursitis or tendinopathy) may not show noticeable changes on imaging. Nonetheless, imaging can strengthen a thorough evaluation [20].

Plain radiographs are usually unremarkable in soft-tissue shoulder complaints, but they can identify gross issues like fractures, intrinsic bone pathology, bony deformations, calcifications, and dislocations.

**Musculoskeletal ultrasound imaging** is a dynamic tool that enables the visualization of tendon movements during shoulder motion [21]. A clinician can detect the supraspinatus tendon slide under the acromion when the patient attempts abduction. Ultrasound can be used to determine if a patient is truly not activating the muscle (when combined with electromyography) [22]. If a patient claims they cannot initiate abduction (suggesting a complete tear or nerve palsy), yet ultrasound clearly shows the supraspinatus tendon intact [23] and no visible attempt at contraction, it indicates a lack of effort rather than true paralysis. If malingering, the patient might not contract a muscle (to pretend weakness). Still, subtle clues, such as co-contraction of other muscles or momentary twitches, could potentially be detected on ultrasound. Ultrasound imaging also aids in ruling out real pathology (e.g., showing an intact rotator cuff when one would expect a tear if the arm were truly pseudoparalytic from injury).

**Magnetic resonance imaging (MRI)** provides detailed views of rotator cuff tendons, labrum, bursae, and other related structures. If a patient reports severe shoulder pain and an inability to move the shoulder, some abnormality on MRI should be expected (e.g., rotator cuff tear, capsular thickening, bone edema from a recent injury). Therefore, an apparently normal radiograph, dynamic ultrasound examination, and MRI in the presence of extreme alleged functional loss raise clinical suspicion. For instance, a patient malingering a rotator cuff tear may have full strength on imaging-based tests: MRI or ultrasound can show intact tendons, and a dynamic ultrasound might even capture them activating muscles despite claimed paralysis. Such discrepancies (normal objective findings versus disabling subjective reports) might indicate secondary gain motives.

A promising development has been the use of **functional MRI (fMRI)** as an objective tool for pain evaluation [20]. Some studies have identified brain activation patterns (neurological signatures) that correspond to experienced pain [24]. The underlying concept is that genuine pain activates specific cerebral regions, such as the anterior cingulate cortex, insula, and secondary somatosensory cortex, as measured by functional magnetic resonance imaging (fMRI), when an individual is exposed to nociceptive stimuli [25]. In contrast, a person simulating pain may not exhibit the same pattern of activation. Although neuroimaging has significantly advanced the understanding of pain processing, it remains neither sufficiently reliable nor practical for determining malingering at the individual level. Nevertheless, research has shown some success in distinguishing feigned vs. actual pain in controlled settings. For example, one study found specific fMRI signatures could predict pain intensity and were absent when subjects were instructed to lie about. Although this technology remains experimental and is not yet integrated into routine clinical assessments, it suggests a future in which brain-based biomarkers could play a supportive role in objectively validating reported pain experiences.

**Infrared thermography (IRT)** is a noninvasive imaging technique that detects infrared radiation emitted from the skin to visualize temperature distribution, thereby highlighting physiological changes in blood flow of underlying tissues. The implementation of infrared thermography in musculoskeletal pain evaluation utilizes mapping of skin temperature distributions, which is suggested to reflect sympathetic nervous system activity and regional blood flow [26]. In genuine painful conditions or nerve injuries, thermography might show abnormal local heat patterns [27]. In shoulder pain disorders, thermography has revealed asymmetrical patterns, either predominantly hypothermic or, to a lesser extent, hyperthermic, in thermographic imaging [28]. Therefore, it has been suggested that routine thermographic studies, in conjunction with other normal exams, may reduce support for specific peripheral inflammatory or autonomic hypotheses in that setting [29]. But IRT in musculoskeletal pain assessment comes with significant limitations. One major challenge is the variability in study quality and consistency of results [30]. Therefore, the current use of thermography in malingering is limited, i.e., while IRT can reflect sympathetic dysfunction and abnormal blood flow in musculoskeletal pain, it should augment, not replace, clinical judgment and conventional diagnostics.

In cases of shoulder pain, a **diagnostic local anesthetic injection** into the subacromial space or intra-articular space may be used to confirm the source of the pain, when substantial short-term analgesia is achieved [31]. In a malingering context, if a patient is truly in pain, the injection should temporarily relieve pain and improve motion. If there is no relief or limitation persists despite the shoulder being anesthetized, it suggests either a non-shoulder source of pain or feigned symptoms. This type of test links the areas of clinical and imaging (preferably performed under fluoroscopy or ultrasound guidance). Caution is needed: psychological factors can still maintain pain behavior even if tissue pain is removed, so a non-response to local anesthetic injection might also occur in certain functional disorders. However, a positive response (sudden improvement in motion with lower pain intensity) can reveal that pain, if any, was the limiting factor. It is important to note that failure to relieve pain does not exclude a genuine complaint, since the local anesthetic block may have missed the actual pain source [32]. Therefore, although anesthetic shoulder blocks can provide objective evidence of pain relief and support for the presence of genuine pathology, considerable caution should be exercised in interpreting a lack of response as indicative of malingering.

### 3.3. Electromyography and Muscle Activation

Electromyography (EMG) and related measures of muscle activation can offer quantitative insight into whether a patient is truly exerting effort.

**Surface EMG** electrodes can record the electrical activity of major shoulder muscles (deltoid, rotator cuff, trapezius, etc.) during attempts at movement [33]. In a maximal voluntary contraction, to maximize force, the nervous system strongly drives agonists and minimizes co-activation of antagonists. High-torque strategies typically involve high agonist activation with reduced antagonist activity [34]. During submaximal efforts (whether sincere or feigned), the pattern changes; typically, fewer motor units are recruited, or they fire less synchronously. By analyzing the EMG signal parameters (amplitude, frequency content, pattern of bursts, and agonist vs. antagonist activity), a genuine maximal effort can be distinguished from submaximal or feigned efforts [35]. Maximal efforts produce high-amplitude, broad-band EMG (dense interference patterns), whereas submaximal or feigned efforts yield lower-amplitude, sparser EMG with different frequency content and coordination [36,37].

**Isokinetic strength ratios:** Another potentially objective parameter is the ratio of eccentric to concentric muscle action (peak torque) at a given angular velocity (DEC parameter). The DEC is inherently related to muscle activation patterns [9]. Higher DEC values are detected when feigning effort compared to genuine maximal effort, yielding an explicit cutoff that separates feigned vs. maximal effort [38]. The underlying physiological reason is that, typically, muscles behave differently at slow and fast speeds in eccentric vs. concentric modes. Still, when the effort is not maximal, these relationships get distorted arbitrarily. The DEC method showed high sensitivity and specificity (both ~78–88%) and a cut-off value of 0.81 for identifying intentionally submaximal efforts [9,38]. Therefore, force ratio analyses can complement EMG by providing objective numbers to compare against normative data or an individual’s own contralateral limb performance.

**Antagonist co-contraction and patterns:** EMG can also reveal if a patient is subtly opposing their own movements. For instance, when asking for shoulder forward flexion and the EMG shows not only minimal anterior deltoid activation but also significant posterior deltoid or latissimus activation (antagonists of flexion), this indicates active resistance to the movement. In a genuinely painful shoulder, one might see some co-contraction due to pain guarding, but typically not a full antagonist activation that prevents movement altogether. The malingerers sometimes over-act, even contracting antagonists to “lock” a limb. Modern surface EMG can detect such patterns and provide visual proof [39]. Markedly lower EMG amplitude on the “affected” side in the absence of neurological injury could indicate incomplete effort.

It should be noted that EMG testing must be performed carefully; generally, nerve conduction studies (NCS) and EMG can rule out true neuropathies (such as axillary nerve palsy or cervical radiculopathy) that could cause real weakness [40]. Because needle EMG sensitivity and specificity for cervical neural lesions are 50–71% and 87–100%, respectively, normal NCS/EMG studies should indicate the absence of an organic cause for shoulder muscle weakness [40].

Taken together, EMG and related analyses of muscle activation offer objective insight into neuromuscular effort that patients cannot volitionally manipulate. When incorporated into the assessment, EMG can document whether a given muscle exhibits an activation pattern physiologically commensurate with the claimed level of effort. Using multiple EMG-derived indices, such as signal amplitude, frequency characteristics, and agonist–antagonist activation patterns, can substantially strengthen the inference of feigned or non-organic weakness when these measures are consistently abnormal in the context of otherwise normal neurophysiological findings.

### 3.4. Motion Capture and Kinematic Analysis

Motion capture technology uses specialized gait analysis, inertial sensors, and photo camera systems. These tools precisely quantify shoulder movement. In detecting feigned restriction, motion capture offers two main advantages: (1) it provides objective measurements of angles, speeds, and movement patterns, and (2) it allows analysis of movement outside of the traditional exam setting.

Markerless motion capture can track joint positions without requiring body-mounted reflective markers [41]. Thus, it can objectively document exactly how far a patient moves their shoulder during various tasks, including even subtle motions. If a patient later claims a different capability, the recorded data can be referenced. Moreover, some markerless systems can run continuously or be used in a patient’s environment.

Small wearable sensors containing gyroscopes and accelerometers (Inertial Measurement Units—IMUs) can be placed on the arm and torso to measure shoulder movement. With this approach, daily activities can be logged. An IMU might record the occasional arm during normal activities that exceed the restricted shoulder range of movement claimed by the examined person [42].

Three-dimensional motion capture allows high-resolution quantification of shoulder and upper-limb kinematics, including joint angles, angular and linear velocity profiles, intersegmental coupling, and path deviations. In healthy individuals performing point-to-point reaching, hand trajectories are typically smooth, with approximately bell-shaped tangential velocity profiles, reflecting an efficient underlying motor control strategy and consistent with optimal control and minimum-variance or related models of reaching dynamics [43]. Motion-capture studies in shoulder pathology show that painful or structurally impaired shoulders often exhibit slower movements, reduced peak velocities, altered timing of peak velocity, and increased variability across repeated trials, capturing aspects of movement quality that are not apparent from static range-of-motion or isometric strength testing alone [44,45].

In clinical or medicolegal contexts, such kinematic deviations may arise from multiple mechanisms, including genuine protective pain behavior, altered neuromuscular control, or deliberate under-performance. For example, individuals with symptomatic rotator cuff tears demonstrate lower maximal elevation, reduced maximum angular velocity, earlier peak velocity, and greater variability than patients with tendinopathy, despite similar pain scores, suggesting that dynamic parameters are sensitive to differences in functional capacity beyond pain alone [45]. Systematic reviews of optical motion capture applied to shoulder disorders conclude that these systems can provide objective, reproducible information on movement speed, timing, and coordination that complements clinical scales and questionnaires, while also emphasizing the need for standardized protocols and further clinical validation [46]. Importantly, there is published data on a specific kinematic feature that has been validated as a stand-alone test and can clearly detect malingering. Therefore, motion-analysis findings must be interpreted in conjunction with the whole clinical picture, in line with broader evidence that no single physical-exam maneuver objectively confirms malingering.

Special attention is required to study the scapulo-humeral rhythm (SHR), which provides a specific framework for analyzing scapulo-humeral coordination. SHR expresses scapulothoracic upward rotation and glenohumeral elevation. In asymptomatic subjects, three-dimensional studies report characteristic patterns of scapular upward rotation, posterior tilt, and external rotation with arm elevation, with an average glenohumeral-to-scapulothoracic ratio of roughly 2:1 across mid elevations. However, the ratio varies by plane of elevation and phase (elevation vs. lowering) [46]. Detailed electromagnetically tracked analyses show that in healthy individuals, the ratios between humeral elevation and scapular motions (upward rotation, tilt, internal/external rotation) change systematically over elevation intervals (e.g., 30–60°, 60–90°, 90–120°), reflecting a highly organized and repeatable scapulothoracic contribution to global arm motion [46].

In contrast, patients with shoulder pathology (e.g., rotator cuff tears, impingement, scapular dyskinesis) often display increased scapular upward rotation, altered posterior tilt, abnormal timing of scapular motion relative to humeral elevation, and greater movement variability, frequently accompanied by compensatory trunk lean or shoulder girdle elevation [44,45]. Such compensatory strategies can result in “paradoxical” patterns in which large scapulothoracic excursions and trunk motions substitute for limited glenohumeral movement. Motion capture and electromagnetic tracking can quantify these phenomena by measuring humeral elevation and scapular rotations, and by computing SHR ratios over the movement arc.

From a conceptual standpoint, in a patient claiming severe restriction of shoulder elevation, kinematic analysis may reveal disproportionate scapular elevation (shrugging) or axial trunk rotation used to move the hand in space, with relatively small changes in actual glenohumeral elevation. Serial assessments might show that when the individual is less focused on the shoulder or when secondary gain contingencies diminish, velocity profiles become more typical and scapular–humeral coupling approaches patterns seen in healthy subjects. This pattern of reversible, context-dependent normalization would support a non-organic component to the limitation. However, current evidence does not establish validated threshold values of SHR that reliably distinguish feign from organic restriction. At present, motion capture should be viewed as an objective tool for documenting and comparing movement patterns, not as a definitive test for malingering [45].

**Marker-based three-dimensional motion capture** provides gold-standard kinematic data. This can precisely measure if a patient’s active ROM differs from passive. Additionally, such systems can detect compensatory motions; for example, instead of actual glenohumeral abduction, a malingerer might laterally flex the trunk to simulate an arm raise. The motion capture would separate those components and show the arm hardly moved relative to the body. In contrast, the body leaned, something that a simple goniometer measurement might miss if one is not careful. Practical factors still limit the adoption of motion capture in routine malingering evaluations. While these systems are promising, their reliability is inconsistent for complex, multi-directional movements [42]. Shoulder motion, involving three degrees of freedom and scapulothoracic movement, can be complex to measure accurately, especially for rotations. Errors or variability in measurement could be misinterpreted as patient inconsistency. Also, some malingerers, if aware of being recorded, might simply rigidly limit all motions consistently (which some can do if they are well-coached or very determined). In such cases, the motion capture will just confirm a limited ROM without necessarily proving the capacity for more. Thus, these tools are best used in conjunction with surprise requests that catch the patient off guard.

Thus, motion capture and kinematic analysis add a powerful objective dimension to evaluating shoulder movement. They are efficient at documenting what the patient does in high precision. When discrepancies arise between recorded capability and claimed limitations, it strongly supports the presence of feigning. As technology becomes more portable, these methods should play an increasingly important role in routine assessments, potentially even before a formal malingering impression is considered.

### 3.5. Artificial Intelligence and Emerging Technologies

The use of artificial intelligence (AI) methods is expected to enhance the detection of malingering. AI can process complex data, e.g., kinematic patterns, facial expressions, or psychometric responses, aiming to find patterns indicative of deception or exaggeration that may elude human observers. For example, in evaluating neck “whiplash” injuries, the study reported 86% sensitivity and 84% specificity for the validity of clinical claims related to this type of injury [47], demonstrating that AI could discern subtle differences in how malingerers move. These systems detect correlates of pain expression, not intent. The AI approach can be translated to shoulder assessments. Although there is currently no clinical evidence on the use of this type of AI for shoulder complaints, an AI algorithm could analyze time series of shoulder joint angles during various tasks. Features such as speed profiles, pauses, tremor, range used, and smoothness can be input into an AI classifier trained on data from genuine patients and instructed malingerers. Future research is essential to promote this approach.

Pain often reveals itself in facial expressions. There is evidence that AI outperforms human clinicians in detecting pain via facial micro-expressions, with an F1-score of 0.8660, indicating high sensitivity to true positives and a balanced precision–recall performance in that dataset [48]. This has direct relevance to malingering, i.e., a person who is genuinely in pain will exhibit involuntary micro-expressions (like winces, grimaces, tightening around the eyes, etc.) that are hard to fake perfectly. Conversely, a malingerer might exaggerate expressions (over-act) or sometimes forget to maintain a painful look when distracted. AI can analyze videos of a patient’s face during physical exam maneuvers or functional tasks to assess whether their facial responses align with genuine pain behavior. For example, if during a shoulder range test the patient stops at a certain angle claiming pain, an AI analyzing their face might note an incongruent lack of expected pain expression or a very conscious, delayed “grimace” that does not match typical timing. While a skilled clinician might notice such a response, AI offers objective analysis and can process subtleties quantitatively [48]. Some **multimodal AI** approaches integrate facial analysis with other biosignals (heart rate, voice stress, etc.) to improve lie detection in pain complaints, achieving a precision of 92%, a recall of 90%, and a specificity of 95% [49,50].

Accordingly, it is foreseeable that a comprehensive system could combine movement data analysis with facial expression and even voice tone. In practice, such technology could be used during independent medical exams: the entire session is recorded and later analyzed by software for inconsistencies between the patient’s verbal claims, facial expressions, and physical performance.

Basically, AI-based methods must be implemented with ethical safeguards and complete transparency. They are intended to support, rather than supplant, clinical judgment, and any algorithmic “malingering score” would require rigorous validation to minimize false-positive classifications. Despite these caveats, current developments suggest that AI will substantially improve our capacity to objectify inherently subjective phenomena such as pain and effort, by summarizing them into quantifiable signals and recognizable patterns. At present, most of these technologies remain in the research domain or are used in highly specialized assessments. Still, it is expected that, following future standardization, they might be incorporated into routine evaluations, including those conducted for rehabilitation, insurance compensation, or military purposes.

AI-based approaches are promising but are currently limited by dataset bias, overfitting, and a lack of external validation, because most reported performance derives from curated datasets and experimental paradigms (including non-shoulder analogs) rather than from representative shoulder claimant populations [47,48,49,50]. More broadly, diagnostic-accuracy evidence shows that spectrum effects and selection and verification bias can increase apparent performance when samples are highly selected or when reference standards are imperfect. These constraints are directly relevant to AI in medicolegal settings [51,52]. Therefore, at present, AI outputs should be regarded as adjunctive and investigational, supporting triangulation of pain and effort representations but not as stand-alone, shoulder-specific tools for malingering determination or medicolegal admissibility without prospective external validation and transparent error-rate reporting [10,12].

### 3.6. Self-Report and Ancillary Tools

Some ancillary methods and contexts do not fit precisely into the categories above but have been used to detect feigned disabilities.

**Specific questionnaires** commonly used in musculoskeletal rehabilitation have versions or subscales to detect inconsistent answers. The Örebro Musculoskeletal Pain Questionnaire (OMPSQ), for example, is a screening tool for psychosocial risk factors in chronic pain [53]. Research supports the finding that feigners show distinct patterns and have developed cut-off scores on the short-form OMPSQ that differentiate real from simulated pain, with an Area Under Curve (AUC) of 0.75–0.84 [54]. Similarly, pain drawings, in which patients shade areas of pain on a body diagram, incorporate validity indicators and have shown an accuracy of about 68.9% for discriminating malingering in pain presentations. Digital body-map drawings of shoulder pain show high test–retest reliability (above 0.90) for area and radiation extent in patients with shoulder disorders [55]. Extremely unusual or non-anatomic pain distributions may raise suspicion of exaggeration or psychological overlay, although they are not definitive [56]. Nonetheless, pain drawings and their validity indices are insufficient as standalone measures for diagnosing feigning or malingering and should always be interpreted within a broader clinical and psychometric framework.

### 3.7. Military vs. Civilian Considerations

Although the fundamental principles of malingering detection apply across settings, military and civilian contexts differ meaningfully in motivational drivers, institutional constraints, and practical implementation, warranting tailored evaluation approaches.

In civilian settings, secondary gain typically involves financial incentives, such as workers’ compensation, personal injury settlements, extended paid leave, or avoidance of occupational duties. These cases often involve fragmented oversight among treating physicians, insurers, and legal representatives. In military environments, motivations may include avoiding deployment, strenuous physical duties, or training requirements, as well as pursuing early discharge or medical separation. Critically, malingering constitutes a punishable offense under military law, potentially increasing the sophistication of deceptive presentations as servicemembers attempt to evade detection.

Military populations exhibit a paradoxical dual phenomenon: while some servicemembers exaggerate symptoms to escape duty, underreporting of genuine injuries is far more prevalent, with studies indicating that nearly half of musculoskeletal injuries go unreported to avoid appearing weak or unfit [5]. This anti-malingering culture complicates evaluation, as medical officers must remain vigilant for both feigned disability and concealed pathology. Additionally, malingering in military personnel frequently coexists with untreated psychological distress, such as deployment-related anxiety or personal crises, necessitating concurrent mental health evaluation rather than purely punitive approaches.

The controlled military environment offers certain evaluative advantages: servicemembers can be observed during physical training, and collateral information from commanding officers regarding functional performance outside clinical settings is often accessible, while surveillance opportunities are rarely available in civilian practice. Conversely, civilian evaluations more frequently require formal medicolegal documentation for litigation or insurance determination, placing greater emphasis on defensible, objective evidence that can withstand legal scrutiny.

Despite contextual differences, the core detection methodology remains consistent: structured clinical examination for inconsistencies, objective biomechanical and neurophysiological testing, appropriate imaging correlation, and psychological assessment for secondary gain indicators. Both settings increasingly emphasize objective documentation over subjective clinical impression alone [20]. The biopsychosocial framework proves valuable across contexts, recognizing that symptom exaggeration frequently clusters with genuine psychosocial burden rather than representing pure fabrication [4].

Thus, while contextual factors shape how and when detection methods are deployed, the underlying diagnostic toolkit remains fundamentally consistent across military and civilian practice.

## 4. Discussion

Detecting feigned restriction of shoulder movement for secondary gain is a complex yet increasingly tractable challenge. Significant progress has been made in developing objective, evidence-based methods to supplement clinical judgment (Table 2). Clinicians no longer need to rely solely on a general impression that a patient is not exerting full effort; they can employ quantitative tools like isokinetic dynamometry, motion capture analysis, and even AI algorithms to gather concrete data. Studies have shown that techniques such as the DEC strength ratio test and frequency content analysis of effort can reliably distinguish genuine maximal performance from feigned or submaximal effort [9]. Likewise, a multidisciplinary approach incorporating imaging, EMG, and symptom validity testing provides a more holistic view, making it harder for malingering to go undetected.

Despite these advances, it is essential to remember that no single test is reliable and that malingering exists on a spectrum. Some patients may partially exaggerate real symptoms, while others may entirely fabricate impairment. The detection strategies must therefore be applied in combination and interpreted in context. A patient might produce consistent efforts on isokinetic testing but still exhibit telling inconsistencies elsewhere (e.g., normal MRI, contradictory behaviors). Although a variety of techniques have been developed for malingering identification, there is currently no standardized, rigorous protocol that addresses the heterogeneous presentations of malingering, especially in shoulder disabilities. Therefore, there remains an essential need to continue research to refine these methods and, importantly, to integrate them into a coherent assessment battery.

Reported sensitivity, specificity, and cut-off values for effort-validity metrics (e.g., DEC ratios), EMG-derived indices, and AI-based classifiers should be interpreted cautiously because they are derived predominantly from experimental models, most commonly instructed-feigning designs or highly selected samples with imperfect reference standards. As such, these estimates describe discrimination under controlled conditions and may overstate performance in routine medicolegal practice, where presentations are heterogeneous (true pathology with pain behavior, partial exaggeration, fear-avoidance, functional symptoms) and where spectrum, selection, and verification biases are likely. Accordingly, these quantitative thresholds are presented in this review as supportive validity indicators that can strengthen inference when they converge with other independent findings, but they should not be used as stand-alone criteria or extrapolated directly to determinations of “simulation” without prospective validation in representative claimant populations and standardized protocols.

For the practical clinical purposes, in suspected feigned shoulder limitation, a stepwise approach should improve consistency and documentation according to the following logical approach (Figure 1): (1) definition of the medicolegal context and record baseline functional observations followed by a structured examination focused on reproducible plausibility checks (non-anatomic patterns, internal inconsistencies, distraction and/or automatic function, and, when feasible, by serial reassessment); (2) correlation of the claimed deficit with structural credibility using appropriate imaging and, when indicated, a targeted diagnostic injection to test pain-limited restriction; (3) quantification of performance with objective measures (dynamometry/isokinetic testing with effort indices, and motion analysis/IMUs where available), using EMG/NCS selectively to exclude neuropathic weakness or corroborate activation patterns; and (4) integration of all data using a convergence model that explicitly considers alternative explanations (pain mechanisms, fear-avoidance or illness behavior, etc.) and reports conclusions as graded, evidence-linked statements rather than single-test determinations.

A key limitation in this field is the lack of a true “gold standard” test for malingering. Intentional deception for external incentives cannot be directly measured, so it is usually inferred from indirect evidence, such as instructed feigning studies, expert case reviews, symptom validity tests, or administrative or legal outcomes [10,12]. As a result, reported diagnostic accuracy varies by setting and study design and is prone to bias. Spectrum bias can occur because many studies compare two extremes: healthy volunteers giving maximal effort versus participants instructed to feign, while real medicolegal cases often fall in the middle (e.g., genuine pathology with symptom amplification, functional neurological symptoms, fear-avoidance), where distinctions are less clear [51,52]. Verification bias is also common because advanced tests (dynamometry, EMG, motion capture, specialized imaging) are often ordered primarily for “suspicious” cases, thereby enriching the sample tested and potentially distorting sensitivity and specificity estimates. Therefore, these tools are best used as supporting evidence within a carefully documented assessment, not as stand-alone proof of malingering.

From a practical standpoint, the approach to a suspected feigned shoulder restriction should be stepwise: start with a thorough clinical evaluation, noting any non-organic signs or inconsistencies; if suspicion remains, perform objective tests such as dynamometry or motion analysis, correlating with imaging and EMG to rule out actual pathology. It is essential to consider psychological evaluation for secondary gain indicators. Documentation should be detailed and factual, focusing on the discrepancies rather than accusatory language.

Because malingering (“simulation”) is defined as the intentional production or exaggeration of symptoms for external incentives, it is a medicolegal conclusion rather than a lesion-based medical diagnosis, and it cannot be established by any single clinical sign or technical test [1,2,10,12]. Accordingly, clinicians should express any opinion in graded, evidence-linked terms and avoid asserting intent as a fact; a defensible formulation is that the findings are inconsistent with expected anatomic and/or physiologic patterns and are most consistent with probable symptom exaggeration or feigned restriction in the presence of external incentives, while explicitly noting that intent cannot be measured directly and that alternative explanations must be considered [1,2,10,12]. The report should then list the concrete basis for that inference: reproducible discrepancies between claimed limitation and observed function (including inconsistency across tasks or context), interpreted with the caution that “non-organic” signs may reflect psychosocial distress or functional neurological symptom presentations rather than deception [4,7,13,16]; results of a structured shoulder assessment with attention to the known limits of commonly used provocative tests in explaining severe disability [6]; and, where performed, objective evidence of submaximal effort from shoulder-specific isokinetic protocols, reported as an effort-validity concern rather than “proof” of malingering [9]. Conceptually, this applies a triangulation framework that integrates subjective reports with replicated discrepancies, credibility relative to pathology, and objective measures, while recognizing that there is no gold standard and that diagnostic-accuracy evidence is vulnerable to spectrum and verification bias in medicolegal populations [10,12,51,52].

### Prospects for Future Research

The continued evolution of technology promises even more sophisticated tools. AI and big data analytics could provide clinicians with decision support. For example, analyzing all data from a clinical case offers a likelihood of malingering relative to a population dataset. However, these will augment rather than replace the clinician’s role, as understanding the patient’s context and potential motives, and explaining inconsistencies, remain essential.

Overall, recent developments described above improved our armamentarium for detecting feigned shoulder movement limitations. Currently, there is an ability to approach such evaluations with a scientific, systematic strategy that protects those who are genuinely injured while exposing those who seek unmerited gain. This ultimately not only benefits payers and systems by reducing fraud but also upholds fairness and ensures that medical resources and empathy are directed at those in genuine need.

## Figures and Tables

**Table 1:** Conceptual distinctions relevant to shoulder disability assessments.

Entity	Intentionality	External Incentives	Practical Clinical Implications (Reporting Focus)
Malingering (“simulation”)	Intentional production or gross exaggeration by definition [1,3]	Present; external incentive is defined [1,2]	Medicolegal inference (not a medical diagnosis). Prefer factual documentation of objective discrepancies and context; avoid categorical accusations without convergent evidence [1].
Symptom magnification/exaggeration	May be intentional or unintentional; intent often uncertain [1,2]	May be present but not defining [2]	Use as a descriptive term for overstatement; do not treat as proof of malingering; document findings and consider alternative explanations [1,2].
Abnormal pain response (APR)/non-organic pain behavior	Not determined (observational phenotype) [4]	Not defining; described in compensable shoulder injury cohorts [4]	Signals disproportionate or incongruent pain behavior and higher psychosocial burden in some cohorts; does not establish deception; prompts careful documentation and further evaluation [4].
Functional disorders (e.g., conversion-type presentations)	Distinguished from malingering by the absence of intentional deception in the clinical framing [3]	Not defining [3]	Requires differentiation from malingering in reports; do not equate inconsistency with deception; document examination findings and diagnostic reasoning [3].

**Table 2:** Summary of detection approaches with evidence level, advantages/limitations, and reported quantitative performance.

Approach (Domain)	Tools/Examples	Typical Level of Evidence	Advantages	Key Limitations	Reported Quantitative Performance (95% CI When Available)	Key Refs.
Medicolegal framing and documentation	Definitions, base rates, ethics, structured reporting	V	Sets defensible scope; supports evidence-linked wording	Not diagnostic; depends on the quality of observations	Base-rate estimates of malingering/symptom exaggeration reported in medicolegal samples (e.g., ~29–30% in some contexts); CIs vary and are not consistently provided	[1,2,3,10,12]
Clinical inconsistency profiling (first-line)	Observation, distraction, functional motor signs, serial exams; APR phenotype	III–IV	Rapid triage; identifies reproducible discrepancies	Non-organic signs are not synonymous with deception; observer bias; requires careful replication/documentation	Hoover’s sign (functional vs. organic leg weakness in suspected stroke): sensitivity ~63%, specificity 100% (CIs not reported)	[4,7,13,14,15,16]
Quantified strength and effort validity	Dynamometry; repeat-trial variability (CV); isokinetic effort indices (DEC paradigm)	II–III (isokinetic/DEC); III–IV (CV generalization)	Converts effort into numbers; contralateral comparison; repeatability; may detect intentionally submaximal torque patterns	Effort-dependent; thresholds not universal; pain/fear can mimic low effort	MMT prediction error ~24% (no CI reported). CV cut-off 15%: sensitivity 0.55, specificity 0.74 (CIs not reported). DEC/isokinetic indices: sensitivity/specificity ~78–88% with cut-off 0.81 (CIs not reported)	[9,17,18,38]
Structural plausibility and pain-source corroboration	XR, dynamic US, MRI; diagnostic anesthetic injections; adjunct research (fMRI signatures, IRT)	II–III (US/MRI/injections); III–IV (fMRI/IRT)	Confirms/excludes structural explanations; supports plausibility testing; blocks can test pain-mediated restriction	Normal imaging does not exclude pain; blocks can miss target/central sensitization; adjunct imaging not validated for individual malingering decisions	Quantitative accuracy varies widely by test and target diagnosis; not consistently comparable across studies/settings (CIs often heterogeneous/not extractable at review level)	[20,21,22,23,24,25,26,27,28,29,30,31,32]
Neurophysiology (effort and exclusion of neuropathy)	Surface EMG amplitude/spectrum; agonist-antagonist patterns; NCS/needle EMG	II–III	Physiological verification can exclude major neuropathic weakness	Signal quality/task design; pain guarding can mimic co-contraction; normal EDX reduces but does not eliminate organic causes	Needle EMG for cervical neural lesions: sensitivity 50–71%, specificity 87–100% (ranges; CIs not provided in manuscript text)	[33,35,36,37,40]
Kinematics and movement quality	3D/optical capture; markerless capture; IMUs; velocity/smoothness; SHR/coupling metrics	II–III	Objective movement documentation; detects compensation/context dependence	No validated kinematic threshold confirms feigning; scapulothoracic measurement is challenging	Shoulder markerless/IMU studies largely report validity/reliability metrics that vary by protocol/device; no diagnostic cut-point established for feigning (CIs not applicable)	[41,42,43,44,45,46]
AI-assisted analytics (emerging)	ML classifiers; facial pain detection; multimodal biosignals	III–IV	High-dimensional pattern recognition: potential standardization	Shoulder-specific medicolegal validation is limited; it detects proxies (pain/effort), not intent; bias/overfitting risk	Whiplash fraud detection SVM (analog domain): sensitivity 86%, specificity 84% (CIs not reported). Facial pain AI: F1-score 0.8660 (CI not reported). Multimodal pain systems: precision 92%, recall 90%, specificity 95% (CIs not reported)	[47,48,49,50]
Self-report validity indicators	OMPSQ feigning-spectrum cut-scores; pain drawings/digital pain maps	III–IV	Adds psychosocial/credibility signals; supports triangulation	Not diagnostic; coached responding; modest accuracy	OMPSQ feigning-spectrum cut scores: AUC 0.75–0.84 (CIs not reported). Pain drawings: accuracy ~68.9% (CI not reported). Digital shoulder pain maps: test–retest reliability > 0.90 (CI not reported)	[53,54,55,56]

Abbreviations: APR: abnormal pain response; CI: confidence interval; CV: coefficient of variation; DEC: dynamic eccentric-to-concentric ratio; EDX: electrodiagnostics; EMG: electromyography; fMRI: functional MRI; IMU: inertial measurement unit; IRT: infrared thermography; MMT: manual muscle testing; MRI: magnetic resonance imaging; NCS: nerve conduction studies; OMPSQ: Orebro Musculoskeletal Pain Questionnaire; ROM: range of motion; SHR: scapulohumeral rhythm; US: ultrasound; XR: radiograph. Levels of Evidence [57]: I: systematic review/meta-analysis of high-quality randomized trials. II: lesser-quality randomized trials or prospective comparative studies. III: retrospective comparative studies or diagnostic studies with important design limitations. IV: case series and non-comparative observational evidence. V: expert opinion, narrative reviews, or mechanistic/physiological rationale without direct comparative clinical evidence.

## Data Availability

No new data were created or analyzed in this study.
